# Obesity, malnutrition, and the prevalence and outcome of hypertension: Evidence from the National Health and Nutrition Examination Survey

**DOI:** 10.3389/fcvm.2023.1043491

**Published:** 2023-03-02

**Authors:** Heng-Zhi Zhang, Yi-Han Wang, Ying-Lin Ge, Shu-Yu Wang, Jin-Yu Sun, Lu-Lu Chen, Shuang Su, Ying Sun

**Affiliations:** ^1^Department of Burn and Plastic Surgery, Children’s Hospital of Nanjing Medical University, Nanjing, China; ^2^The First Clinical Medicine College, Nanjing Medical University, Nanjing, China; ^3^Department of Biomedical Engineering, College of Medicine, Tianjin University, Tianjin, China; ^4^Department of Cardiology, The First Affiliated Hospital of Nanjing Medical University, Nanjing, China; ^5^Department of Anatomy, Histology, and Embryology, Nanjing Medical University, Nanjing, China; ^6^Nanjing Pukou Central Hospital, Pukou Branch Hospital of Jiangsu Province Hospital, Nanjing, China

**Keywords:** hypertension, obesity, malnutrition, waist circumference, double burden of malnutrition

## Abstract

**Background:**

Nutritionally unhealthy obesity is a newly introduced phenotype characterized by a combined condition of malnutrition and obesity. This study aims to explore the combined influence of obesity and nutritional status on the prevalence and outcome of hypertension.

**Methods:**

Participants collected from the National Health and Nutrition Examination Survey (NHANES) database were divided into four subgroups according to their obesity and nutritional conditions, as defined by waist circumference and serum albumin concentration. The lean-well-nourished was set as the reference group. Logistic regression models were applied to evaluate the hypertension risk. Kaplan–Meier analysis and Cox proportional hazard regression models were used to assess the survival curve and outcome risk of participants with hypertension.

**Results:**

A total of 28,554 participants with 10,625 hypertension patients were included in the analysis. The lean-malnourished group showed a lower hypertension risk (odds ratio [OR] 0.85, 95% confidence interval [CI]: 0.77–0.94), while the obese-well-nourished condition elevated the risk (OR 1.47, 95% CI: 1.3–1.67). Two malnourished groups had higher mortality risks (HR 1.42, 95% CI: 1.12–1.80 and HR 1.31, 95% CI: 1.03–1.69 for the lean and obese, respectively) than the reference group. The outcome risk of the obese-well-nourished group (HR 1.02, 95% CI: 0.76–1.36) was similar to the lean-well-nourished.

**Conclusion:**

Malnutrition was associated with a lower risk of developing hypertension in both lean and obese participants, but it was associated with a worse outcome once the hypertension is present. The lean-malnourished hypertension patients had the highest all-cause mortality risk followed by the obese-malnourished. The obese-well-nourished hypertension patients showed a similar mortality risk to the lean-well-nourished hypertension patients.

## 1. Introduction

Arterial hypertension is a leading risk factor for multiple cardiovascular diseases (CVDs) and renal disability (1). About 10 million deaths globally can be attributed to hypertension each year (2, 3). Despite the relatively stable global average blood pressure in these decades, the prevalence of hypertension is continuously increasing in various low- and middle-income regions (1, 4).

Due to the unhealthy diet and behavior patterns, obesity, a condition strongly associated with type 2 diabetes and CVDs, has become a growing worldwide health problem (5). However, obesity is a phenomenon of high heterogeneity and can occur under a broad spectrum of metabolic situations (6). Interestingly, malnutrition and obesity can be observed simultaneously in individuals as part of the so-called “double burden of malnutrition” (7, 8). This hybrid condition of malnutrition and obesity is recognized to be highly implicated with the inflammatory state and the risk of non-communicable diseases (7).

Recently, an intriguing research on heart failure patients revealed that the obese-malnourished participants had significantly higher comorbidity burden and less favorable cardiac outcomes compared with other nutrition and obesity statuses (9). This result led to our speculation about the potential influence of this phenotype on hypertension. Although obesity has long been identified as an important risk factor for hypertension (10), the relationship between nutrition status and blood pressure remains vague (11, 12). To our knowledge, no previous research has investigated the combined effect of nutrition and obesity status on hypertension. In this study, we aim to explore the association of nutrition status defined by serum albumin (SA) levels and abdominal obesity with the prevalence and outcomes of clinical hypertension.

## 2. Methods

### 2.1. Data source and study population

The National Health and Nutrition Examination Survey (NHANES) is a multistage health survey based on interviews and physical/laboratory examinations of the civilian US population. The National Death Index (NDI) is a centralized death record information database collecting the follow-up information from the date of medical examination to either death or censoring (December 31, 2015). This study was based on the publicly available data of 7 consecutive NHANES cycles (2001–2002, 2003–2004, 2005–2006, 2007–2008, 2009–2010, 2011–2012, 2013–2014) and the NDI database. The demographic data, body measurements, blood pressure, CVD, smoking/drinking status, medical conditions, standard biochemistry profiles, income, and education levels of participants were extracted. The race of participants was categorized as non-Hispanic white, non-Hispanic black, Mexican American, other Hispanic, and Other. The exclusion criteria were as follows: (1) participants aged <18 or > 80 years, (2) without body mass index (BMI) or waist circumference records, (3) without blood pressure records, (4) pregnant individuals, (5) diagnosed with cancer, (6) deceased within 3 months. After selecting patients with hypertension, 10,625 participants were finally enrolled. National Center for Health Statistics Research Ethics Review Board approved the analysis, and informed consent was acquired from all individuals.

### 2.2. Study definitions

Abdominal obesity was defined by waist circumference, with a cut-off value of 102/88 cm for males and females, respectively, as proposed by the National Cholesterol Education Program-Adult Treatment Panel III (NCEP-ATP III) (13). Nutrition status was defined by the serum albumin concentration as in the previous study (9). Participants with SA < 45 g/L or ≥ 45 g/L were recognized as malnourished or well-nourished.

Each participant’s blood pressure was measured following the American Heart Association standardized protocol three times after resting 5 minutes in a seated position. Hypertension was defined as previously described (14, 15): (1) average systolic blood pressure ≥ 130 mmHg or diastolic blood pressure ≥ 80 mmHg, (2) self-reported hypertension, or (3) self-reported administration of anti-hypertensive medications.

The income levels of participants were evaluated by family income-to-poverty ratios (PIRs), which was calculated as the ratio of family income to the federal poverty level. PIR was categorized as <1.33, 1.33–<3.50, and ≥ 3.50, following the qualification criterion for the US federal Supplemental Nutrition Assistance Program (16).

### 2.3. Covariates

Multiple covariates related to hypertension were assessed in this study to minimize bias. To be specific, age, sex, body mass index (BMI), race (non-Hispanic white, non-Hispanic Black, Mexican American, other Hispanic, and other races), diabetes, taking prescriptions for hypertension, smoking, alcohol drinking, and education level of the participants were collected from the NHANES and were adjusted in the statistical analysis.

### 2.4. Statistical analysis

The statistical reporting recommendations by the American Heart Association were followed in the study (17). Participants were categorized into four groups according to their waist circumference and nutrition status. The group with low waist circumference and fine nutrition status was selected as the reference group (the lean-well-nourished group). Kolmogorov–Smirnov test was used to assess the normality. Continuous variables with normal distribution were provided as mean ± standard deviation, while skewed distributed variables were provided as median with interquartile range. Categorical variables were reported as percentages. ANOVA test, Kruskal-Wallis test, and chi-square test were adapted for comparing the baseline characteristics of continuous variables with normal and skewed distribution, and categorical variables, as appropriate.

Logistic regression models were applied to evaluate the association of obesity and malnutrition with the prevalence of hypertension, and Cox proportional hazard regression models were used to assess the association of obesity and malnutrition with all-cause death in participants with hypertension. Odds ratios (ORs) and hazard ratios (HRs) with 95% confidence intervals (CIs) were calculated in the logistic and Cox regression analyses, respectively. In either logistic or Cox regression analysis, unadjusted analysis was performed at first. Then two different adjusted models were performed to minimize the bias caused by covariates. Model 1 was adjusted for age, sex, race, diabetes, having CVDs, taking prescriptions for hypertension, smoking/drinking status, and education level. Model 2 was adjusted for model 1 covariates plus BMI. An additional age-stratified analysis for the young (below 45 years old) and the elder (above 45 years old) were then conducted with the same method.

Moreover, we further assessed the association between obesity and nutrition status with the prognosis of hypertension by Kaplan–Meier survival analysis. Pairwise comparisons of the survival rates in different groups were performed by the log-rank test. A two-tail value of *p* < 0.05 was considered statically significant. All statistical analyses were performed using the R software (version 3.6.1; R Foundation for Statistical Computing, Vienna). An additional age-stratified analysis for the young (below 45 years old) and the elder (above 45 years old) were then conducted with the same method.

## 3. Results

### 3.1. Baseline characteristics

The demographics, cardiovascular health status, cardiovascular risk factors, and behavioral factors of the participants were provided in Table 1. A total of 28,554 participants were included in the study, including 10,625 (37.2%) participants diagnosed with hypertension. During a median follow-up of 6.8 years, 2,162 (7.6%) deaths were observed, including 376 (17.3%) CVD deaths.

**Table 1:** Baseline characteristics of the study population.

	Lean-well-nourished^*^ (*n* = 5,133)	Lean-malnourished (*n* = 8,104)	Obese-well-nourished (*n* = 2,679)	Obese-malnourished (*n* = 12,638)	*p* value
Age	35.0 [26.0;48.0]	45.0 [32.0;60.0]^#^	47.0 [35.0;61.0]^#,†^	51.0 [39.0;64.0]^#,†,&^	<0.001
Sex (Male/Female), *n* (%)	3,806/1,327 (74.1/25.9)	4,634/3,470 (57.2/42.8)^#^	1,587/1,092 (59.2/40.8)^#,†^	4,389/8,249 (34.7/65.3)^#,†,&^	<0.001
Hypertension (No/Yes), *n* (%)	4,113/1,020 (80.1/19.9)	5,883/2,221 (72.6/27.4)^#^	1,464/1,215 (56.4/45.4)^#,†^	6,469/6,169 (51.2/48.8)^#,†,&^	<0.001
Race:					<0.001
Non-Hispanic White	2,405 (46.9%)	3,310 (40.8%)	1,457 (54.4%)	5,492 (43.5%)	
Non-Hispanic Black	711 (13.9%)	1,821 (22.5%)	338 (12.6%)	3,117 (24.7%)	
Mexican American	935 (18.2%)	1,389 (17.1%)	509 (19.0%)	2,423 (19.2%)	
Other Hispanic	397 (7.7%)	678 (8.4%)	225 (8.4%)	1,030 (8.2%)	
Other races	685 (13.3%)	906 (11.2%)	150 (5.6%)	576 (4.6%)	
PIR level:					<0.001
<1.33	1,449 (28.2%)	2,309 (28.5%)	723 (27.0%)	4,004 (31.7%)	
1.33–3.5	1,623 (31.6%)	2,710 (33.4%)	895 (33.4%)	4,335 (34.3%)	
≥3.5	2,061 (40.2%)	3,085 (38.1%)	1,061 (39.6%)	4,299 (34.0%)	
Education level:					<0.001
Below high school	1,148 (22.4%)	2,210 (27.3%)	646 (24.1%)	3,693 (29.2%)	
High School	1,149 (22.4%)	1,756 (21.7%)	660 (24.6%)	3,080 (24.4%)	
Above high school	2,836 (55.3%)	4,138 (51.1%)	1,373 (51.3%)	5,865 (46.4%)	
CVD (No/Yes), *n* (%)	4,940/193 (96.2/3.8)	7,514/590 (92.7/7.3)	2,461/218 (91.9/8.1)	11,131/1,507 (88.1/11.9)	<0.001
Mortality status (No/Yes), *n* (%)	4,938/195 (96.2/3.8)	7,427/677 (91.6/8.4)	2,515/164 (93.9/6.1)	11,512/1,126 (91.1/8.9)	<0.001
CVD death (No/Yes), *n* (%)	5,105/28 (99.5/0.5)	7,984/120 (98.5/1.5)	2,648/31 (98.8/1.2)	12,441/197 (98.4/1.6)	<0.001
SBP	117.3 [109.3;127.3]	118.0 [108.7;130.0]^#^	124.0 [114.0;135.3]^#,†^	122.7 [112.7;136.0]^#,†^	<0.001
DBP	70.7 [64.0;78.0]	70.0 [62.7;77.3]^#^	74.0 [65.3;81.7]^#,†^	72.0 [64.0;80.0]^#,†,&^	<0.001
WC	86.1 [79.1;93.6]	86.0 [80.0;94.3]^#^	105.8 [99.4;112.0]^#,†^	107.0 [99.0;116.2]^#,†,&^	<0.001
BMI	24.0 [21.7;26.3]	24.2 [22.0;26.5]^#^	30.4 [28.1;33.4]^#,†^	31.9 [28.8;36.2]^#,†,&^	<0.001
Total-to-HDL ratio	3.5 [2.8;4.5]	3.4 [2.8;4.4]^#^	4.3 [3.4;5.3]^#,†^	4.0 [3.2;4.9]^#,†,&^	<0.001
Triglycerides	101.0 [68.0;158.0]	98.0 [68.0;150.0]^#^	149.0 [99.0;232.0]^#,†^	133.0 [90.0;199.0]^#,†,&^	<0.001
Diabetes (No/Yes), *n* (%)	4,818/315 (93.9/6.1)	7,315/789 (90.3/9.7)	2,274/405 (84.9/15.1)	9,653/2,985 (76.4/23.6)	<0.001
HbA1c	5.3 [5.1;5.5]	5.4 [5.1;5.6]^#^	5.5 [5.2;5.8]^#,†^	5.6 [5.3;6.0]^#,†,&^	<0.001
FPG	89.0 [83.0;96.0]	90.0 [83.0;98.0]^#^	93.0 [86.0;103.0]^#,†^	95.0 [87.0;109.0]^#,†,&^	<0.001
eGFR	109.5 [89.8;130.8]	99.3 [77.7;122.4]^#^	125.2 [96.3;159.1]^#,†^	123.3 [91.4;161.0]^#,†^	<0.001
Smoking (No/Yes), *n* (%)	2,816/231 7 (54.9/45.1)	4,245/3,859 (52.4/47.6)	1,435/1,244 (53.6/46.4)	6,932/5,706 (54.9/45.1)	0.003
Drinking (No/Yes), *n* (%)	4,694/439 (91.4/8.6)	7,155/949 (88.3/11.7)	2,387/292 (89.1/10.9)	2,387/292 (89.1/10.9)	<0.001

^*^Obese and nutrition status was defined by waist circumference and serum albumin concentration with cut-off values of 102 (males)/88 (females) and 45 g/L, respectively. *p*-value < 0.05 for post hoc comparison against ^#^lean-well-nourished, ^†^lean-malnourished, and ^&^obese-well-nourished. Continuous variables were all skewed distributed and therefore provided as median with interquartile ranges. Categorical variables are provided as percentages. PIR, family income-to-poverty ratio; CVD, cardiovascular disease; SBP, systolic blood pressure; DBP, diastolic blood pressure; WC, waist circumference; BMI, body mass index; HDL, high-density lipoprotein; HbA1c, hemoglobin A1c; FPG, fasting plasma glucose; eGFR, estimated glomerular filtration rate.

Among four groups classified by abdominal obesity and nutrition status, the obese-malnourished group had the most participants (12,638, 44.3%), much higher than the obese-well-nourished group (2,679). The obese-malnourished group was generally the oldest (median 51 years) and had the highest BMI (median 31.9), drinking percentage (16.2%), and crude prevalence of diabetes (23.6%). The obese-malnourished group also presented the highest crude prevalence of hypertension (48.8%). The obese-malnourished group had the largest absolute number (8,249) and proportion (65.3%) of females. Being the opposite of the obese-malnourished, the lean-well-nourished group presented the youngest age (median 35 years), lowest alcohol drinking proportion (8.6%), and lowest crude prevalence of diabetes (6.1%) and hypertension (19.9%).

### 3.2. The association of obesity and malnutrition with the prevalence of hypertension

Table 2 shows the results of the logistic regression models assessing the association of obesity and malnutrition with the prevalence of hypertension. In the multivariable-adjusted model 1, compared with the lean-well-nourished group, both two obese groups showed a significantly higher risk of hypertension, with ORs of 2.29 (95% CI: 2.04–2.57) and 1.94 (95% CI, 1.77–2.13) respectively for the well−/malnourished. Interestingly, the old lean-malnourished group had a significantly lower risk for hypertension (OR 0.83, 95% CI: 0.73–0.95) compared with the reference group, but the significance was not observed in the younger participants.

**Table 2:** The association of obesity and malnutrition with the prevalence of hypertension.

	Unadjusted		Model 1^*^		Model 2^**^	
	OR (95% CI)	*p*	OR (95% CI)	*p*	OR (95% CI)	*p*
Total						
Lean-well-nourished	Reference		Reference		Reference	
Lean-malnourished	1.52 (1.4–1.66)	<0.001	0.89 (0.81–0.98)	0.017	0.85 (0.77–0.94)	0.001
Obese-well-nourished	3.35 (3.02–3.71)	<0.001	2.29 (2.04–2.57)	<0.001	1.47 (1.3–1.67)	<0.001
Obese-malnourished	3.85 (3.56–4.15)	<0.001	1.94 (1.77–2.13)	<0.001	1.07 (0.96–1.19)	0.252
Age below 45						
Lean-well-nourished	Reference		Reference		Reference	
Lean-malnourished	1.07 (0.92–1.24)	0.380	0.99 (0.85–1.16)	0.921	0.95 (0.81–1.11)	0.537
Obese-well-nourished	3.16 (2.66–3.74)	<0.001	2.87 (2.4–3.43)	<0.001	1.76 (1.45–2.13)	<0.001
Obese-malnourished	2.98 (2.63–3.4)	<0.001	2.75 (2.37–3.19)	<0.001	1.38 (1.15–1.66)	<0.001
Age above 45						
Lean-well-nourished	Reference		Reference		Reference	
Lean-malnourished	1.03 (0.92–1.16)	0.568	0.83 (0.73–0.95)	0.005	0.82 (0.72–0.93)	0.002
Obese-well-nourished	2.09 (1.81–2.41)	<0.001	1.89 (1.62–2.21)	<0.001	1.29 (1.1–1.53)	0.002
Obese-malnourished	2.22 (1.99–2.48)	<0.001	1.62 (1.43–1.83)	<0.001	0.97 (0.84–1.11)	0.662

OR, Odds ratio; CI, Confidence interval. ^*^Model 1: Adjusted for age, sex, race, diabetes, having cardiovascular diseases, taking prescriptions for hypertension, smoking/drinking status, and education level. ^**^Model 2: Adjusted for covariates in model 1 plus body mass index.

In model 2, which adjusted covariates in model 1 plus BMI, a significant elevation of hypertension risk was still observed in the obese-well-nourished group (OR 1.47, 95% CI: 1.3–1.67). The older lean-malnourished group still showed a lower risk (OR 0.82, 95% CI: 0.72–0.93) comparing with participants with normal waist circumference and nutrition status. However, the significant rise in hypertension risk in the obese-malnourished group was only observed in younger participants (OR 1.38, 95% CI: 1.15–1.66) in model 2.

### 3.3. The association of obesity and malnutrition with the outcome of hypertension patients

**Figure 1:**
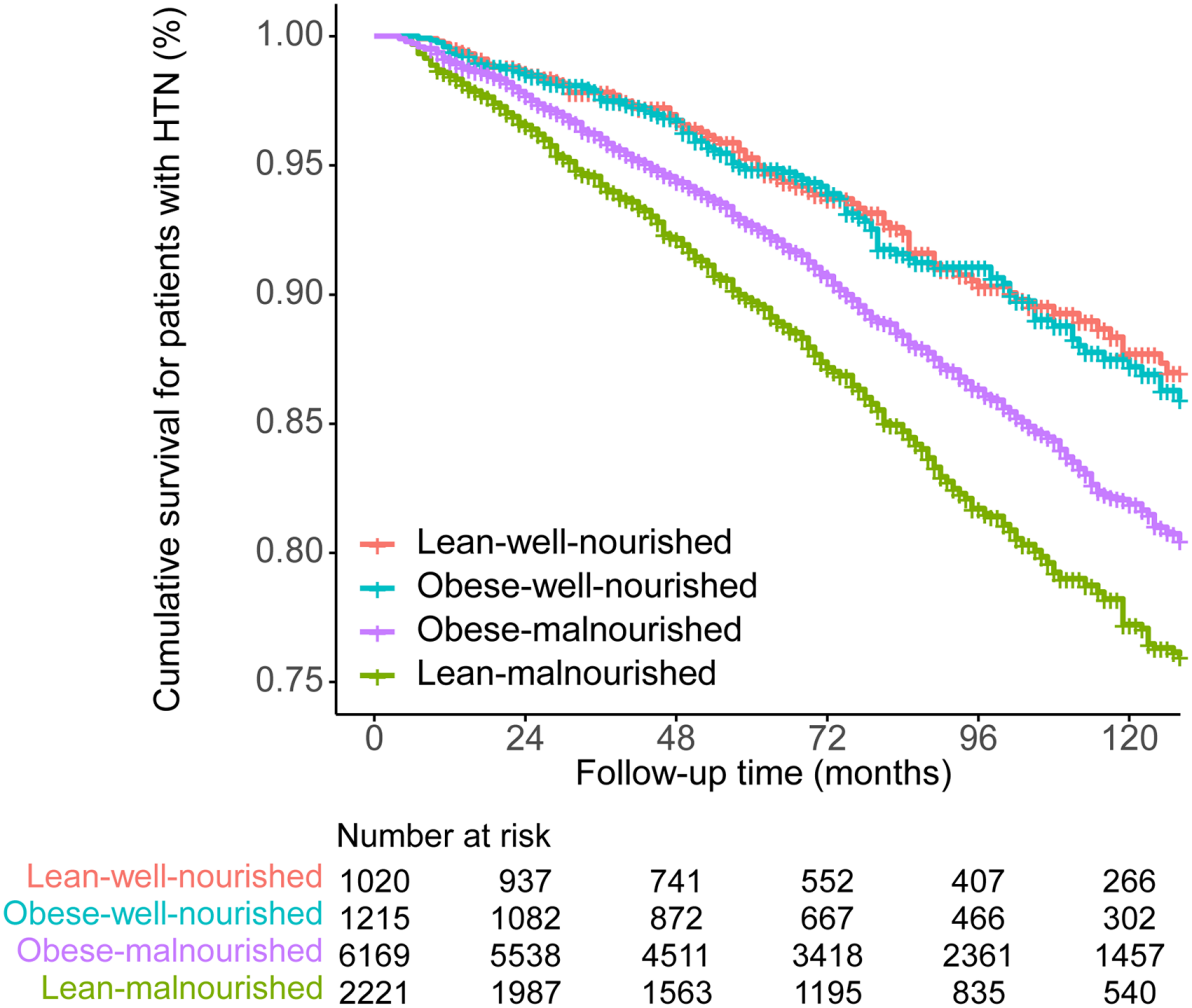
Kaplan–Meier survival curve of the four groups divided by obesity and nutrition status.

Among 10,625 participants with hypertension, 1,370 (12.9%) deaths were observed during the follow-up. Figure 1 and Table 3 show the results of the Kaplan–Meier analysis for evaluating the association of obesity and malnutrition with the risk of all-cause death in hypertension patients. Compared with the lean-well-nourished group, both malnourished groups showed significantly elevated death risk. Interestingly, the difference between the two malnourished groups is also statistically significant, with the lean-malnourished having a higher mortality risk. However, no significant difference was observed between the lean-well-nourished and the obese-well-nourished groups.

**Table 3:** Pairwise comparisons of different groups in the Kaplan–Meier survival analysis.

	Lean-well-nourished	Lean-malnourished	Obese-well-nourished
Lean-malnourished	<0.001	–	–
Obese-well-nourished	0.290	<0.001	–
Obese-malnourished	<0.001	<0.001	0.001

Two-tail *p*-values were calculated via log-rank test.

We then examined the survival of hypertension participants in each group by multi-adjusted Cox models to eliminate the bias caused by cardiovascular covariates (Table 4). In model 1, compared with the lean-well-nourished, only the elevation of mortality risk in the lean-malnourished group reached significance (HR 1.42, 95% CI: 1.12–1.79). After further adjustment of BMI in model 2, the unstratified analysis found a significantly higher risk in the lean-malnourished group (HR 1.42, 95% CI: 1.12–1.80) and obese-malnourished group (HR 1.31, 95% CI: 1.03–1.69). In the age-stratified model 2, the lean-malnourished group had higher outcome risks (HR 2.61, 95% CI 1.10–6.18 and HR 1.36, 95% CI 1.06–1.74 for the young and the elder, respectively), and the risk elevation of the obese-malnourished group also nearly reached statistical significance (HR 1.29, 95% CI 1.00–1.67).

**Table 4:** The association of obesity and malnutrition with all-cause death in participants with hypertension.

	Unadjusted		Model 1^*^		Model 2^**^	
	HR (95% CI)	*p*	HR (95% CI)	*p*	HR (95% CI)	*p*
Total						
Lean-well-nourished	Reference		Reference		Reference	
Lean-malnourished	2.06 (1.63, 2.61)	<0.001	1.42 (1.12, 1.79)	0.004	1.42 (1.12, 1.80)	0.004
Obese-well-nourished	1.16 (0.87, 1.53)	0.307	0.92 (0.69, 1.22)	0.568	1.02 (0.76, 1.36)	0.908
Obese-malnourished	1.61 (1.29, 2.01)	<0.001	1.15 (0.92, 1.45)	0.223	1.31 (1.03, 1.69)	0.031
Age below 45						
Lean-well-nourished	Reference		Reference		Reference	
Lean-malnourished	2.64 (1.13, 6.18)	0.025	2.61 (1.10, 6.18)	0.029	2.61 (1.10, 6.18)	0.029
Obese-well-nourished	1.85 (0.70, 4.86)	0.212	1.66 (0.63, 4.37)	0.309	1.61 (0.58, 4.45)	0.361
Obese-malnourished	2.16 (0.97, 4.81)	0.058	1.63 (0.70, 3.79)	0.252	1.57 (0.60, 4.06)	0.356
Age above 45						
Lean-well-nourished	Reference		Reference		Reference	
Lean-malnourished	1.67 (1.31, 2.13)	<0.001	1.35 (1.06, 1.73)	0.017	1.36 (1.06, 1.74)	0.015
Obese-well-nourished	0.96 (0.71, 1.28)	0.77	0.88 (0.66, 1.18)	0.401	0.98 (0.72, 1.33)	0.901
Obese-malnourished	1.27 (1.00, 1.60)	0.048	1.12 (0.88, 1.42)	0.344	1.29 (1.00, 1.67)	0.052

HR, Hazard ratio; CI, Confidence interval. ^*^Model 1: Adjusted for age, sex, race, diabetes, having cardiovascular diseases, taking prescriptions for hypertension, smoking/drinking status, and education level. ^**^Model 2: Adjusted for covariates in model 1 plus body mass index.

## 4. Discussion

Nutritionally unhealthy obesity, or the individual-level double burden of malnutrition, is a newly introduced phenotype characterized by a combined condition of malnutrition and obesity, caused mainly by poor food quality and inadequate micronutrient consumption (9, 18). Traditionally, the double burden of malnutrition is thought to affect primarily low- to middle-income countries (7, 8). However, our study observed an individual-level double burden of malnutrition (low SA levels plus high waist circumference) in a considerable proportion of US participants, suggesting the long-standing importance of promoting favorable diet and health behaviors not only in developing countries but still in developed ones. We found that the demographic distribution of the obese-malnourished was typically older and often women, which is generally consistent with a recent study on Asian participants (9). This demographic distribution pattern is similar to that of malnutrition alone, the underlying mechanisms of which may include socioeconomic burden and metabolic features of aging (19).

Our results suggested that the association between obesity and the all-cause mortality risk of hypertension patients is modified by nutrition status. The obese-malnourished hypertension patients have a significantly higher mortality risk than the lean-well-nourished, whereas the risk of the obese-well-nourished is comparable with that of the reference group. This result implies that malnutrition may be an outcome indicator of hypertension which is independent of obesity. Similarly, a newly published study on heart failure patients reported that compared with the obese-well-nourished, the obese-malnourished status has a higher outcome risk, higher likelihood of comorbidities, and notable cardiac remodeling (9). Malnutrition in obese individuals is often overlooked in clinical practice, but recent evidence, including our study, suggested the significant association between this phenotype and the prognosis of CVDs and other diseases (20, 21).

This study further enhanced the understanding of the value of the SA test in clinical practice. Our results suggested that SA test can be an easy and inexpensive test in clinical practice to assist in evaluating the prognosis of hypertension patients. Beyond our findings, low SA levels have also been found to be an indicator of the emergence and worsening of some CVDs (22), and an independent predictor of ischemic heart disease and ischemic stroke even after adjustments for BMI, liver functions, and kidney functions (23). The underlying mechanisms of the association of low SA levels and cardiovascular dysfunctions are ambiguous. Speculations are that it is related to the inflammatory condition in malnutritional status with low SA. As human albumin is the most copious antioxidant in the whole blood, the insufficiency of SA will initialize oxidative stress and inflammation, which play a key role in the pathogenesis of hypertension and multiple CVDs (22, 24). At the same time, low SA levels can be the result of various causes including kidney diseases and general organ dysfunction due to CVDs (25), which may directly affect or reflect the cardiovascular health condition.

Among the malnourished hypertension participants, we found that obese individuals had a better outcome than the lean. This phenomenon is related to the so-called obesity paradox, i.e., obesity is associated with a higher risk for developing CVDs, but a better prognosis if the disease is present (26). Recent evidence argued that this paradox might stem more from the poorer catabolic reserve and more cachexia of the lean group rather than the potential benefit of the obesity itself, as studies have confirmed that low body fat percentage and low BMI are independent predictors of worse outcomes of CVDs (27, 28). Other possible causes for the better outcome of obese individuals may include younger age at presentation, lower prevalence of smoking, lower levels of atria natriuretic peptide, and more usage of cardiac medications (26).

Our study suggested that malnutrition may reduce the prevalence of hypertension. After being stratified by obesity status, the two malnourished groups presented a lower risk for developing hypertension than their well-nourished counterparts. This result is partly in line with earlier observations that SA level is positively associated with blood pressure (12, 29). However, this result does not imply that weakening nutritional condition is a desirable way to prevent hypertension since malnutrition is a harmful state associated with various comorbidities and remarkably higher mortality risk (30, 31). We speculate that this result is related to the SA’s function of maintaining colloid osmotic pressure, but precise underlying causes are worthy of further research.

Body mass index remains hitherto the most widely used measure to report obesity and related cardiovascular risk. However, since only weight and height are considered, BMI cannot comprehensively describe the heterogeneous composition of body weight and fat distribution within the obese population (32). Compared with BMI, waist circumference can better indicate visceral adiposity and provide additive information (33, 34), and was therefore recommended as a vital sign in clinical practice according to a recent consensus (35). Previous studies have reported that when analyzed in the same model, waist circumference was a risk factor for CVDs, but BMI was usually found to be a neutral or even protective factor (36–38). It is a strength that we used waist circumference to define obesity and adjusted for BMI in the analysis to fully investigate the potential value of obesity and nutrition status in evaluating the risk of hypertension.

### 4.1. Limitations

First, on the association between obesity and nutrition status with the prognosis of hypertension, this study only explored the risk of all-cause mortality without confirmation of the causal relationship. There remains the possible existence of multiple confounding factors that should not be ignored. Low SA levels may result from different causes such as chronic diseases and inflammatory conditions, many of which are directly related to worse cardiac outcomes, so the precise causal chain still needs to be further identified. Second, the baseline waist circumference and SA level of participants may change during the follow-up, but we cannot analyze the possible influence of these changes on the outcome. Third, the nutritional status was only evaluated by SA levels in this study. However, SA levels were affected by concurrent health issues apart from nutrition status. Therefore, further validation based on other nutritional indicators should be performed in the following study. Fourth, hypertension defined by both examination and self-report, may affect the robustness of the results. Lastly, due to the limitation of the data source, this study could not distinguish between primary and secondary hypertension.

## 5. Conclusion

This study based on NHANES observed the existence of an individual-level double burden of malnutrition in US citizens. We found that the association between obesity and the prognosis of hypertension is altered by nutrition status. Malnutrition defined by SA was associated with a lower risk of developing hypertension in both lean and obese participants, but it was associated with a worse outcome if the hypertension is present. The lean-malnourished hypertension patients had the highest all-cause death risk followed by the obese-malnourished. The obese- and lean-well-nourished hypertension patients showed a similar mortality risk lower than the mal-nourished.

## Data availability statement

The original contributions presented in the study are included in the article/supplementary material, further inquiries can be directed to the corresponding authors.

## Author contributions

H-ZZ, J-YS, SS, and YS conceived and designed the study. Y-LG, S-YW, J-YS, and L-LC analyzed the data. H-ZZ, Y-HW, and Y-LG wrote the paper. All authors provided critical revisions of the manuscript and approved the final manuscript.

## Funding

This study was supported in part by the Natural Science Foundation of Jiangsu Province (No. 21KJB320006) and the College Students Innovation and Entrepreneurship Training Program of Jiangsu Province (No. 202110312006Z and No. 202210312010Z).

## Conflict of interest

The authors declare that the research was conducted in the absence of any commercial or financial relationships that could be construed as a potential conflict of interest.

## Publisher’s note

All claims expressed in this article are solely those of the authors and do not necessarily represent those of their affiliated organizations, or those of the publisher, the editors and the reviewers. Any product that may be evaluated in this article, or claim that may be made by its manufacturer, is not guaranteed or endorsed by the publisher.
